# Adaptive Hierarchical Evidence Fusion for Sensitive Field Detection in Structured Data: A Gated Residual Correction Network

**DOI:** 10.3390/e28060582

**Published:** 2026-05-22

**Authors:** Junpeng Hu, Xiao Guo, Jinan Shen, Minghui Zheng

**Affiliations:** 1 School of Cyber Science and Engineering, Sichuan University, Chengdu 610065, China; 2021326240017@stu.scu.edu.cn; 2 College of Intelligent Science and Engineering, Hubei University for Nationalities, Enshi 445000, China; 202430330@hbmzu.edu.cn (X.G.); shenjinan@hbmzu.edu.cn (J.S.)

**Keywords:** structured data, sensitive field detection, cross-domain generalization, adaptive feature fusion, gated residual network

## Abstract

Automatic detection of sensitive fields in structured data is a critical prerequisite for privacy compliance and data governance. However, existing approaches face severe cross-domain generalization challenges. Hand-crafted pattern rules often fail under highly heterogeneous naming conventions, while single statistical models tend to overfit and degrade sharply under distribution shifts between training and deployment domains. These limitations stem from the weak semantic signals and distributional heterogeneity of structured data, which make it difficult to simultaneously capture explicit rules and latent, variant-sensitive attributes. To address these challenges, we propose a detection framework based on multi-view complementary features and a Hierarchical Gated Residual Network (HGRN). The framework first constructs a full-spectrum feature system that integrates explicit rules and implicit statistical fingerprints (e.g., entropy and character texture) to fill the semantic gap. It then introduces a decision mechanism combining robust priors with dynamic residual calibration: a random forest provides a stable probabilistic anchor, which is further nonlinearly corrected by a learnable gating-and-expert network. This design explicitly resolves the cognitive conflict between rule-dominated regions and complex distributional regions. Experiments on multiple real-world datasets—including DeSSI, CMS Open Payments and Home Credit—show that the proposed method achieves a Macro-F1 of 0.9408 on DeSSI and exhibits strong in-domain performance. Under strict frozen-model cross-domain transfer, HGRN mitigates the catastrophic collapse observed in pure neural baselines and maintains moderate detection capability, offering interpretable trust allocation between rule-based priors and data-driven correction in both financial and healthcare scenarios.

## 1. Introduction

With the deepening of digital transformation, data has become a key production factor alongside land, labor, capital, and technology [1]. In critical infrastructures such as financial risk control, smart healthcare, and e-government, the flow and reuse of massive structured datasets are generating substantial economic value. To ensure secure and orderly data circulation, regulatory regimes worldwide are tightening. The EU General Data Protection Regulation (GDPR), China’s Data Security Law, and sectoral standards all emphasize the establishment of data classification and grading schemes. Within this compliance framework, sensitive field identification in structured data is the foundation and prerequisite for implementing such classification. Only after accurately locating database attributes that carry personal privacy or critical business information can downstream tasks such as access control, differential privacy, and data masking be meaningfully applied [2].

In practice, the accuracy of sensitive field detection directly determines the effectiveness of classification-and-grading governance. Insufficient detection capability leads to governance failures: on one hand, over-detection causes non-sensitive fields to be misclassified as sensitive, introducing unnecessary masking and access restrictions, increasing operational costs, and impeding business flows [3]. On the other hand, false negatives mean highly sensitive fields become effectively “invisible” to the security stack, escaping access control and audit, and forming “dark data” channels. Such blind spots are a root cause of unauthorized cross-border data transfers, insider misuse, and large-scale data breaches [4,5]. Building an automatic detection mechanism that accurately covers both explicit and implicit sensitive information and can adapt across complex business domains is thus key to moving data classification and grading from “policy on paper” to “technology in practice”.

Despite the urgency, sensitive field detection in structured data still faces fundamental challenges in both academia and industry, most notably in cross-domain generalization. Unlike natural language data with rich contextual semantics, structured data typically exhibits two properties [6]:

- Weak semantics. Field names are often abbreviations (e.g., c_id), codes, or system-specific identifiers, which severely limits the usefulness of lexical-semantic matching.
- Distributional heterogeneity. The same type of sensitive field (e.g., user IDs) can have markedly different value distributions (entropy, character patterns) and structural roles across systems (e.g., DeSSI’s synthetic environment vs. healthcare-specific CMS data).

Most existing methods rely either on static rule libraries or single-view statistical models, making them brittle in dynamic, heterogeneous feature spaces [7]. When deployed beyond the training domain, these models lack the ability to adaptively reweight heterogeneous cues and are easily overfitted or underfitted, causing significant drops in recall and rendering them unsuitable for automated, high-recall classification and grading.

To tackle these issues, we propose a Hierarchical Gated Residual Network (HGRN) for sensitive field detection. Instead of relying purely on semantics or rules, we design a six-dimensional multi-view feature system that spans explicit rules and implicit distributional fingerprints, and we combine it with a two-level “robust prior + dynamic correction” architecture to achieve adaptive evidence fusion for heterogeneous structured data.

The main contributions are:

(1) A detection framework for sensitive-field identification that explicitly targets the governance trade-off between over-detection and missed detection, validated through extensive multi-dataset experiments.
  The framework is built on a “robust prior + dynamic calibration” architecture designed to mitigate performance collapse under distribution shift. We evaluate it on three real-world datasets—DeSSI, CMS Open Payments, and Home Credit—where it achieves Macro-F1 scores of 0.9408, 0.8164, and 0.8535 in in-domain settings, respectively. Under strict frozen-model cross-domain transfer, the framework exhibits controlled degradation rather than catastrophic collapse, and gating-weight analysis confirms an intelligible balance between rule-based decisions and learned corrections.
(2) A full-spectrum “three-layer” feature representation tailored to weak semantics and distributional heterogeneity in structured data.
  We organize six complementary feature families into three functional layers: (i) an explicit constraint layer (regex pattern matching and token-level header semantics) that serves as weakly supervised anchors for standardized names; (ii) an implicit distribution layer (normalized entropy, character texture, and uniqueness ratio) that acts as the last line of defense for semantically opaque identifiers such as hashed IDs; and (iii) a contextual association layer (mutual information and BERT-based contextual features) that captures quasi-identifiers whose sensitivity emerges only in combination with other fields. This cascading design fills semantic gaps where any single signal would fail.
(3) An adaptive hierarchical fusion mechanism with theoretical guarantees, based on a gated combination of an RF prior and a neural expert.
  At Level 1, a random forest leverages its natural strength on mixed-feature tabular data to provide a stable probabilistic anchor. At Level 2, a lightweight gating network learns sample-wise confidence scores that dynamically scale the contribution of the RF prior against an MLP-based expert correction. We formalize this fusion with an adaptive error bound (Proposition 1) and a cross-domain generalization bound (Theorem 1), showing that freezing the RF prior restricts the effective hypothesis space and promotes stability under distribution shift. The mechanism thus achieves smooth switching between rule-dominated regions and complex distributional regions.

## 2. Related Work

Sensitive field detection in structured data is a foundational step for privacy governance and data security, aiming to identify fields in relational databases, data warehouses, and data lakes that pose privacy risks. Research spans three main paradigms: rule-based methods, machine-learning/statistical methods, and deep learning approaches [8].

Early systems predominantly follow a pattern-driven strategy, using regular expressions and dictionaries to detect explicit sensitive types such as phone numbers, emails, and national ID numbers [9]. These methods are efficient and interpretable, yet fragile under schema heterogeneity and prone to false negatives for implicit sensitive fields lacking stable surface patterns [10].

More recent work emphasizes that privacy risk is context- and relation-dependent: a field benign in isolation can become sensitive when exhibiting strong statistical dependence with identifiers, enabling inference or linkage attacks [11]. To capture such risks, feature-based methods combine value distributions, statistical dependence, and metadata, shifting from single-signal detection to multi-signal modeling [11]. Concurrently, the data management community has developed column representations for semantic type detection even when headers are uninformative. Sherlock [12] uses column values; Sato [13] incorporates table context via topic vectors and CRF; TURL [14] learns Transformer-based table representations from web-scale corpora; and Doduo [15] applies pre-trained language models to column annotation. These models demonstrate that integrating column values with contextual/table-level information yields more robust semantics than name-based heuristics alone. However, they inherently rely on table-level context (neighboring columns, metadata, or full-table serialization) and multi-class type supervision, making them incompatible with single-field, frozen-model cross-domain sensitive-field detection where isolated headers and values are the only available inputs.

At the system level, Aurum [16] integrates schema, value, and relational signals for scalable discovery across heterogeneous datasets, while Starmie [17] strengthens contextualized column embeddings in noisy data-lake metadata. In privacy research, vulnerability analyses of privacy-preserving record linkage show that linkage risks persist even under protection pipelines, motivating risk-oriented assessments beyond surface detection [18].

Beyond the column-annotation paradigm, multi-source information fusion has been studied extensively under uncertainty. The Dempster–Shafer (D–S) evidence theory provides a principled framework for combining conflicting signals without prior probabilities, and has been applied to large-scale multi-source data fusion [19], multi-sensor conflict resolution [20], and multi-view classification [21]. These methods highlight adaptive credibility allocation among heterogeneous sources—an idea aligned with our gated residual mechanism. However, D–S-based approaches typically operate on discrete belief mass assignments and require carefully designed basic probability assignment functions, making them difficult to integrate with continuous neural representations in high-dimensional mixed-feature spaces.

Overall, these efforts converge toward a multi-dimensional feature fusion perspective for sensitive field detection: a unified decision model should integrate pattern-based clues, entity/value semantics, schema similarity, statistical dependence, and distributional priors. It is worth noting that the aforementioned column-understanding methods—Sherlock, Sato, TURL, and Doduo—target semantic type detection rather than binary sensitive-field classification, and their reliance on table-level context and multi-class supervision renders them incomparable under our single-field, frozen-model cross-domain protocol. Consequently, our empirical comparison is restricted to models that naturally accept the same isolated field-level feature representation, so as to isolate the effect of the proposed hierarchical fusion mechanism. Our work follows this line and further contributes a hierarchical gating-and-residual mechanism to resolve the conflict between rule-based and statistical models under cross-domain distribution shifts.

## 3. Preliminaries

This section introduces the technical background underpinning our method: column-level modeling of structured fields, entropy and mutual information from information theory, context-enhanced sequence labeling, and expert–gating frameworks for multi-signal fusion.

### 3.1. Problem Formulation

We formalize sensitive field detection as a supervised binary classification problem over structured relational fields. Consider a source domain Ds comprising Ns field-level samples drawn i.i.d. from an underlying distribution Ps over the field space F and label space Y. Each field f ∈ F is represented as an ordered pair f = (h, $V_{f}$), where $h\in \Sigma^{*}$ denotes the field name (header string) over alphabet Σ, and $V_{f}=\{v_{1},\ldots ,v_{n}\}$ is a multiset of *n* ≥ 1 sampled non-empty values from that column. The ground-truth label y ∈ Y = {0, 1} indicates whether f is non-sensitive (y = 0) or sensitive (y = 1).

A feature extractor $\Phi :F\to X\subseteq {\mathbb{R}}^{d}$ maps each field f to a d-dimensional real-valued feature vector x=Φ(f). In our framework, d = 6, corresponding to the six complementary feature views detailed in Section 5.1. The hypothesis space H consists of all measurable functions $F_{\theta }:X\to [0,1]$ parameterized by θ∈Θ, where $F_{\theta }(x)$ estimates the posterior probability P(y = 1∣f).

Given the binary cross-entropy loss, specifically, as shown in Equation (1):

(1) $$L(F_{\theta }(x),y)=-[y\log F_{\theta }(x)+(1-y)\log(1-F_{\theta }(x))]$$

the learning objective is to minimize the regularized empirical risk over Ds, as shown in Equation (2):

(2) $$\theta^{*}=\arg\min_{\theta \in \Theta }\;\frac{1}{N_{s}}\sum_{i=1}^{N_{s}}L(F_{\theta }(\Phi (f_{i})),y_{i})+\lambda \Omega (\theta )$$

where Ω(θ) denotes a regularization term (e.g., L2 penalty) with hyperparameter λ ≥ 0.

For cross-domain evaluation, let $D_{t}={\left\{(f_{j},y_{j})\right\}}_{j=1}^{N_{t}}$ be a target-domain dataset drawn from $P_{t}$ such that $P_{t}(f,y)\neq P_{s}(f,y)$. The frozen-model transfer protocol (Section 6.6) requires evaluating the fixed model $F_{\theta^{*}}$ on $D_{t}$ without updating the parameters $\theta^{*}$ or re-estimating the feature extractor Φ, thereby isolating the inherent domain-generalization capability from any adaptation effects.

This decoupled formulation—separating feature extraction Φ from decision making $F_{\theta }$—allows us to analyze domain shifts in a modular fashion and motivates the hierarchical fusion architecture introduced in Section 5.

### 3.2. Column-Level Modeling of Structured Fields

In relational databases and data warehouses, the column is the smallest governance and control unit. Compared to the sentence–document hierarchy in natural language, structured fields have the following characteristics:

Strong locality: Field values are typically short strings, codes, or numbers without rich natural-language context.

Observable distributions: Each field consists of many values of the same type; the empirical distribution over these values reflects latent semantic properties (e.g., identifiers vs. categories).

Clear structural roles: Fields often function as primary keys, foreign keys, or business identifiers.

We therefore adopt a column-level modeling paradigm. A field is represented as: $f=(h,V_{f})$.

Where h is the header (field name) and $V_{f}=\{v_{1},\ldots ,v_{n}\}$ is a multiset of sampled values from that column. All feature extraction and classification are performed at the field level, not at the individual value level. This assumption underlies the six-dimensional feature design in Section 5.

### 3.3. Entropy for Characterizing Field Distributions

Shannon entropy quantifies the uncertainty of a discrete random variable and is widely used in structured data analysis to characterize the dispersion and information density of column values.

Let $V=\{v_{1},\ldots ,v_{k}\}$ be the distinct values in a field *f*, with empirical distribution p(v). The formal representation is shown in Equation (3):

(3) $$H(f)=-\sum_{v\in V}p(v)\log p(v)$$

In column modeling:

High-entropy fields tend to have high cardinality and often correspond to identifiers or near-unique tokens.

Low-entropy fields are more likely to be enumerations or status attributes.

Due to the differences in sample size and cardinality among different fields, this paper uses normalized entropy in the feature construction stage to enhance comparability across fields and datasets. The relevant calculation methods will be provided in detail in Section 5.

### 3.4. Mutual Information and Inter-Field Dependence

In multi-column tables, fields often exhibit non-trivial statistical dependencies. Mutual information (MI) captures both linear and non-linear dependence between two random variables and is widely used for feature selection and dependency analysis.

For discrete variables *X* and *Y* with joint distribution P(x,y) and marginals P(x),P(y). The formal representation is shown in Equation (4):

(4) $$I(X;Y)=\sum_{x\in X}\sum_{y\in Y}P(x,y)\log\frac{P(x,y)}{P(x)P(y)},$$

MI is symmetric and non-parametric, making it suitable for quantifying complex dependencies between fields. In our framework, MI serves as a field-level association strength measure to support contextual features.

### 3.5. Context-Enhanced Sequence Labeling and Column Aggregation

Even though structured field values are short, they can still contain entities recognizable by language models. Named entity recognition (NER) treats sequence labeling as mapping an input token sequence $S=(w_{1},\ldots ,w_{L})$ to a tag sequence $Y=(y_{1},\ldots ,y_{L})$ with probability. The formal representation is shown in Equation (5):

(5) p(Y∣S;θ)=i=1∏Lp(yi∣S,θ),

where *θ* denotes model parameters.

Because single field values are short and ambiguous, we use context-enhanced prompt templates and multi-sample aggregation: we combine the header with several sampled values into a natural-language prompt and apply a pre-trained NER model, aggregating its outputs across samples to obtain a stable field-level semantic indicator.

### 3.6. Expert Models and Gating Mechanisms

In tasks involving heterogeneous features or multiple signals, a single model often struggles to maintain stable performance over the entire input space. A common approach is to combine expert models with gating mechanisms.

#### 3.6.1. Expert Networks

Expert models are specialized predictors that perform well in particular regions of the feature space or for particular patterns (e.g., rule-based models, tree ensembles, neural networks). In multi-expert architectures, individual experts serve as candidate sources of judgment; an upper-level fusion mechanism determines how to weight them.

#### 3.6.2. Gating Mechanisms for Dynamic Fusion

Gating mechanisms learn input-dependent weights for experts. Given feature vector x, a gating function produces α(x) ∈ [0, 1], which modulates the contribution of different experts in the final prediction. Compared with static weights, gating enables sample-wise adaptive fusion, improving robustness under conflicting signals and heterogeneous distributions. This paradigm guides our design of the hierarchical fusion structure.

## 4. Problem Formulation and Design Motivation

### 4.1. Problem Definition and Regulatory Background

Data protection regimes worldwide are being rapidly strengthened. GDPR Article 9 designates special categories of personal data—such as racial or ethnic origin, biometrics, and health data—as subject to stricter processing requirements [22]. China’s Data Security Law (Article 21) establishes a data classification and grading protection system [23], and the Personal Information Protection Law (Article 28) further defines sensitive personal information [24]. These regulations share a common assumption: effective governance is conditional on accurate identification of high-risk data elements. Only when sensitive fields are correctly located can access control, privacy-enhancing techniques, and audit trails be properly enforced.

Detection failures create serious risks in real systems. False positives lead to excessive classification of non-sensitive data, unnecessary masking and access restrictions, higher operational costs, and reduced business agility. More critically, false negatives leave highly sensitive fields unmonitored, creating dark data channels that bypass access control and auditing. According to IBM’s 2023 Cost of a Data Breach Report, failures in sensitive data identification are among the fundamental drivers of large-scale breaches in healthcare and financial services, with average incident costs reaching USD 4.45 million. The reliability of sensitive field detection thus forms a central bottleneck in moving from “formal regulation” to “technically enforced governance”.

In response to the above challenges, we formalize sensitive field recognition at the column level based on the representation f = (h, $V_{f}$) defined in Section 3.1. The goal is to learn a binary classifier F(f) ∈ {0, 1} that decides whether a field *f* should be labeled as sensitive (1) or non-sensitive (0).

The core difficulty lies in cross-domain generalization: when the source domain $D_{s}$ and the target domain $D_{t}$ have substantially different feature distributions, the performance of existing methods often degrades sharply. This bottleneck stems from the weak semantics and distributional heterogeneity of structured data, which make it difficult for a single model to simultaneously capture both explicit rules and implicit, variant-sensitive attributes (see Section 4.3 for details).

### 4.2. Capability and Limitations of Existing Methods

Existing techniques for sensitive field detection fall into two main paradigms: rule-based matching and single-model statistical learning. While each is effective in specific settings, both have inherent limitations. Table 1 summarizes typical applicability and failure modes.

These limitations form an overarching rule–statistics cognitive conflict. Rule-based methods exhibit low bias in constrained domains but lack the flexibility to generalize; statistical models are flexible but may overfit and behave unreliably in rule-dominated regions. Conventional fusion schemes (e.g., majority voting, static weighting) typically ignore that different cues have domain-dependent and sample-dependent reliabilities. This conflict motivates both multi-view feature expansion and a hierarchical architecture designed for adaptive, confidence-aware fusion.

### 4.3. Motivation for Multi-View Feature Expansion

Weak semantics and heterogeneous distributions imply that any single signal will be fragile under domain shift. Pattern rules cannot cover all naming variants; purely statistical features may misclassify high-entropy but non-sensitive fields. To fill semantic gaps and improve robustness, we need a full-spectrum feature system covering explicit patterns, implicit distributions, and contextual semantics [25].

Table 2 outlines our six-dimensional feature design, grouped into three functional layers. The explicit constraint layer (regex + header semantics) targets strongly formatted and standardized fields; the implicit distribution layer (statistical fingerprints + uniqueness ratio) captures semantically opaque identifiers; and the contextual association layer (MI + BERT-based semantics) focuses on quasi-identifiers and combination leakage risk. The layers form a cascading set of safety nets.

Our experiments (Section 6.4) show that individual feature dimensions in isolation achieve substantially lower recall than the full six-dimensional fusion, confirming that no single signal suffices for broad sensitive-type coverage.

### 4.4. Hierarchical Adaptive Evidence Fusion

Flat fusion strategies (simple averaging, static weights, or single end-to-end deep models) cannot adequately resolve the rule–statistics conflict: rules are stable but rigid; statistical models are flexible but high-variance. We therefore propose a two-level hierarchical architecture embodying “stable baseline + dynamic correction”:

Level 1 (Evidence Layer): A random forest (RF) trained on the six-dimensional features provides a robust prior probability for sensitivity.

**Figure 1:**
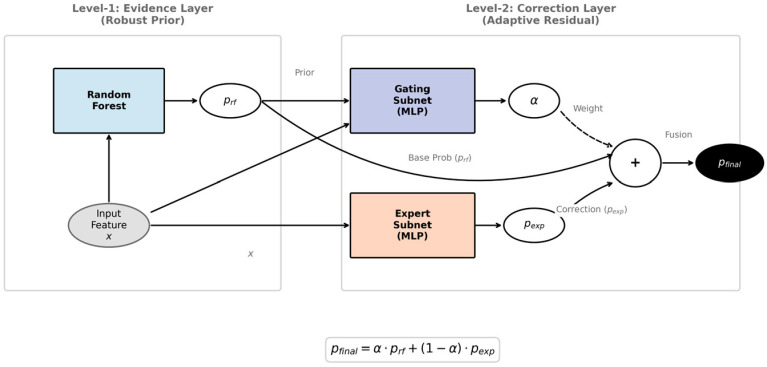
Hierarchical Architecture Diagram. Hierarchical Architecture Diagram. Blue block: Random Forest (RF) prior; purple block: Gating Subnetwork (MLP); orange block: Expert Subnetwork (MLP). Solid arrows indicate forward data flow; dotted arrows indicate probabilistic outputs ($p_{rf}$, $p_{\exp}$, *α*). The plus sign (+) denotes weighted summation of the RF prior and expert correction. The residual fusion formula at the bottom is a schematic illustration; the definitive expression is given in Equation (17).

Level 2 (Correction Layer): A neural gated-residual head—composed of an expert subnet and a gating subnet—learns when to trust the RF prior and when to correct it using additional nonlinear structure in the feature. The specific framework diagram is shown in Figure 1.

This design addresses three central issues:

1. Static rules and fixed weights. Traditional methods cannot dynamically adjust the importance of explicit vs. implicit cues under changing distributions, leading to over-detection or missed detection. Our gating weight α is learned as a function of each sample, enabling adaptive rebalancing between explicit rules and implicit statistics.
2. Cross-domain generalization under distribution heterogeneity. When the same sensitive type exhibits very different distributions across systems, single-view models often fail catastrophically outside the training domain. By learning “when to doubt the prior”, the gate automatically identifies low-confidence regions for the RF and triggers residual correction, mitigating catastrophic degradation under domain shift.
3. Overfitting/underfitting in complex feature spaces. Rich features are necessary to combat weak semantics but easily cause high-variance deep models under limited labels. Using RF as a strong, low-variance baseline constrains the function space; the upper neural network focuses on learning residuals, reducing overfitting while preserving flexibility. This satisfies stringent high-recall requirements in automated classification and grading.

## 5. Adaptive Hierarchical Evidence Fusion Framework

**Figure 2:**
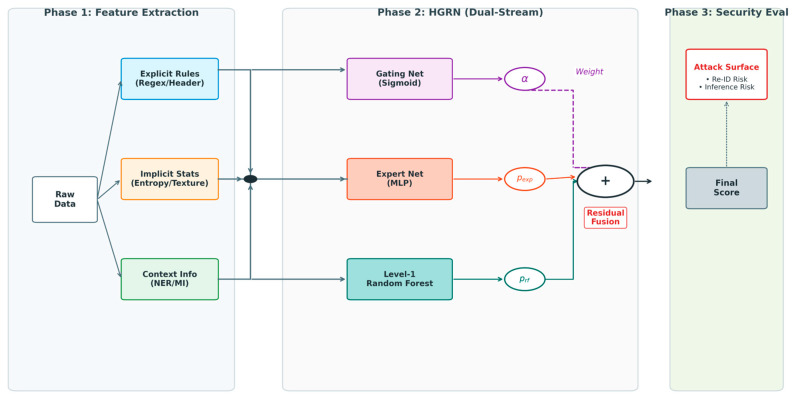
Overall system flow chart. Color coding: blue = explicit-rule features (regex + header semantics); yellow = implicit-distribution features (entropy + texture); green = contextual-association features (MI + NER); purple = gating subnet; orange = expert subnet (MLP); cyan = Level-1 random forest; red = attack-surface audit; gray = final score. Solid arrows indicate forward data flow; dotted arrows indicate probabilistic or control signals ($p_{rf}$, $p_{\exp}$, *α*); the black dot (•) denotes the scalar multiplication operation in the residual fusion; the plus sign (+) denotes weighted summation of the RF prior and expert correction. The detailed fusion formula is given in Equation (17).

To tackle weak semantics, heterogeneous naming, and cross-domain distribution shift, we propose a Hierarchical Gated Residual Network (HGRN) for sensitive field detection. The framework, illustrated conceptually in Figure 2, consists of three tightly coupled stages:

1. Phase 1—Multi-View Feature Extraction: Map each field $f=(h,V_{f})$ into a six-dimensional feature vector $X_{i}\in R^{6}$ that aggregates explicit constraints, implicit distributional cues, and contextual association signals.
2. Phase 2—Hierarchical Evidence Fusion (HGRN): Use a dual-stream architecture to address cross-domain generalization:
  Level 1: an RF model provides a robust prior probability $p_{rf}\left(x\right)$;
  Level 2: a gated expert network produces an expert probability $p_{\exp}\left(x\right)$ and a gating weight α(x), combining them in a residual fashion.
3. Phase 3—Risk Scoring and Decision: The final calibrated probability $p_{final}\left(x\right)$ enables flexible thresholding (default τ = 0.5), and the threshold can be tuned to prioritize recall in high-security scenarios.

### 5.1. Multi-View Feature Construction

To overcome the limitations of single-feature approaches, we designed six complementary feature dimensions grouped into three layers.

#### 5.1.1. Explicit Rules and Metadata Constraints

(1) Regex pattern matching.

We maintain a rule library R of regular expressions for strongly formatted types such as bank card numbers, email addresses, and phone numbers, derived from domain knowledge and regulatory specifications (e.g., GDPR). For a field f with sampled values $V_{f}$, the regex feature is activated if the fraction of values matching any rule r ∈ R exceeds threshold $\tau_{regex}$. The specific definition of regular patterns is shown in Table 3.

(2) Token-level header semantic alignment

Header strings provide global semantic cues even when value semantics are weak or noisy. Simple exact matching is brittle under naming heterogeneity (usr_id, user_phone_no, etc.). We thus propose token-level alignment:

1. Morphological decomposition: Tokenize the header *h* into a sequence of tokens $T_{f}=\{t_{1},t_{2},\ldots ,t_{m}\}$
2. Semantic mapping: Given a domain lexicon *K* organized into sensitive concept categories, we compute the maximum lexical similarity between each token *t* and any lexicon entry under category *c*. We use a normalized Levenshtein distance, denoted Lev(⋅), to handle misspellings and irregular abbreviations. The formal representation is shown in Equation (6):
  (6) $$\mathrm{Sim}(t,C)=\max_{k\in K_{C}}\left(1-\frac{\mathrm{Lev}(t,k)}{\max(|t|,|k|)}\right)\;$$

Ultimately, the semantic prior score $S_{prior}(f)$ of field *f* is defined as the optimal alignment between the morpheme sequence and the sensitive knowledge base. The formal representation is shown in Equation (7):

(7) $$S_{\mathrm{prior}}(f)=\max_{t\in T_{f}}\left(\max_{C\in C_{\mathrm{sens}}}\mathrm{Sim}(t,C)\right)\;$$

This feature, as a weakly supervised signal, can prevent cognitive drift in the model when content features fail.

#### 5.1.2. Implicit Statistical and Distributional Fingerprints

For implicit sensitive attributes such as hashed IDs or random tokens, we construct distributional fingerprints capturing micro-patterns invisible to simple inspection.

(1) Statistical fingerprint features.

This dimension consists of two complementary statistical indicators:

Normalized Shannon entropy: Quantify the information density of field values. Sensitive identifiers typically have high entropy, while categorical attributes have lower entropy values. Based on the definition of information entropy given in Section 3.2, this paper calculates the normalized entropy of the sampling value distribution of each field f to characterize the information density of the field. The formal representation is shown in Equation (8):

(8) $$E_{norm}(f)=\frac{H(f)}{\log_{2}\left|V_{unique}\right|+\epsilon }$$

Character texture vector T(f), capturing the proportion of digits, letters, and special symbols across all sampled characters of the field. The formal representation is shown in Equation (9):

(9) $$T(f)={\left[\frac{N_{digit}}{L},\frac{N_{alpha}}{L},\frac{N_{symbol}}{L}\right]}^{⊤}$$

where *L* is the total number of characters across all samples. Email addresses, for example, show characteristic non-zero special-symbol density (e.g., ‘@’, ‘.’).

These are aggregated (e.g., via a simple mapping function or concatenation) into a statistical fingerprint feature.

(2) Structural stability: uniqueness ratio.

To detect key-like fields without clear semantics, we define the uniqueness ratio. The formal representation is shown in Equation (10):

(10) $$U(f)=\frac{\left|\left\{v|v\in V_{f}\right\}\right|}{N}\times \frac{1}{1+\sigma (\mathrm{len}(V_{f}))}$$

Among them, ∣∣ represents the cardinality of the set and $\sigma (\mathrm{len}(V_{f}))$ is the standard deviation of the field length. When U (f) → 1 and the length variance is extremely small, the maximum probability of this field is the system generated primary key or ID number.

#### 5.1.3. Context-Aware and Associative Semantics

(1) BERT-based contextual semantics.

Standalone short values such as “Smith” can be ambiguous (person vs. organization). Direct NER on isolated values is unreliable. Inspired by prompt learning, we design a contextual prompt:

Let h be the header and $\left\{v_{1},v_{2},\ldots ,v_{k}\right\}$ be up to k = 3 sampled non-empty values. The formal representation is shown in Equation (11):

(11) Prompt(f)=“Column name:h.Examplevalues:v1,v2,…,vk”.

Input the prompt into the pre trained BERT-NER model M to obtain the recognized entity set $E_{f}$. We define a binary indicator feature $S_{bert}(f)$, to mark whether the field has sensitive semantics under strong contextual prompts. The formal representation is shown in Equation (12):

(12) $$S_{\mathrm{bert}}(f)=I\left(\exists e\in E_{f}|\mathrm{Type}(e)\in C_{\mathrm{sens}}\wedge \mathrm{Conf}(e)>\tau \right)$$

Among them, $C_{\mathrm{sens}}=\{PER,LOC,ORG\}$ is the set of sensitive entity categories of interest, and τ=0.6 is the confidence threshold. The advantage of this method is that it utilizes $h_{f}$ as a context anchor, significantly improving the model’s semantic disambiguation ability for short text field values. For example, when $h_{f}=“Owner”$ and the content is “Apple”, the model tends to recognize it as an organization rather than a fruit.

(2) Mutual information as an operational context function.

Unlike the other five passive feature dimensions, MI is integrated as an operational function that actively modulates the fusion mechanism. Let S = {f_j:y_j = 1} denote the set of confirmed sensitive fields in the current table. For a target field X, specifically, as shown in Equation (13):

(13) $$phi\_MI\left(X\right)\;=\;max\_\left\{Y\;in\;S\right\}\;I\_norm\left(X;\;Y\right)$$

where I_norm(X;Y) = I(X;Y)/min(H(X),H(Y)) in [0, 1] is the normalized mutual information from Section 3.4. The output phi_MI(X) serves two operational roles:

(i) Feature-level injection. phi_MI(X) is concatenated into the six-dimensional vector x as the contextual association signal, quantifying combination-leakage risk.
(ii) Gate-level modulation. phi_MI(X) is fed into the gating subnet as a distributional reliability indicator. During end-to-end training, the gate learns to associate high phi_MI(X) with quasi-identifier behavior: when a field exhibits strong statistical dependence with known sensitive fields, the gate suppresses *α*(x) and up-weights the expert correction branch, recognizing that the RF prior (which relies on local field patterns) is insufficient for context-dependent sensitivity. Conversely, when phi_MI(X) is low, the gate retains confidence in the RF prior. This sample-wise adaptive routing transforms MI from a descriptive statistic into an active fusion operand.

### 5.2. Adaptive Fusion via Gated Residual Stacking

We now present the HGRN architecture that fuses these six-dimensional features in a hierarchical manner.

#### 5.2.1. Level 1: Robust Prior via Random Forest

At Level 1, we choose a random forest (RF) as the base learner to obtain a robust prior probability. RF is preferable to a deep network at this level because:

1. It naturally handles mixed continuous and categorical features (entropy, binary flags, counts) with limited sensitivity to feature scaling.
2. It offers strong noise robustness and low variance under limited labeled data, thanks to bagging.

Let the RF consist of K trees $\left\{T_{1},\ldots ,T_{K}\right\}$.The formal representation is shown in Equation (14):

(14) $$p_{rf}(x)=\frac{1}{K}\sum_{k=1}^{K}T_{k}(x)$$

where each $T_{k}\left(x\right)\in \left[0,1\right]$ is the class probability at the leaf node. This serves as a baseline anchor; Level 2 only needs to learn how to correct the RF’s errors rather than learning the entire decision boundary from scratch.

#### 5.2.2. Level 2: Gated Neural Correction

Level 2 comprises an expert subnet and a gating subnet.

Expert subnet. The expert subnet $f_{\exp}:R^{d}\to \left[0,1\right]$ is a multi-layer perceptron (MLP) that captures nonlinear interactions overlooked by RF. The formal representation is shown in Equation (15):

(15) $$h_{1}=\sigma (W_{1}x+b_{1}),h_{2}=\sigma (W_{2}h_{1}+b_{2}),p_{exp}(x)=\sigma ({w_{3}}^{⊤}h_{2}+b_{3})$$

where σ is a nonlinear activation (e.g., ReLU in hidden layers, Sigmoid in the output), and $\left\{W_{i},b_{i}\right\}$ are learnable parameters.

Gating subnet. To decide when RF is reliable, the gate takes as input the concatenation of *x* and $p_{rf}\left(x\right)$: $\tilde{x}=[x;p_{rf}(x)],$ then, the gate control signal is calculated. Its formal representation is shown in formula (16):

(16) $$g=\phi \left(W_{g}\tilde{x}+b_{g}\right),\;\alpha (x)=\sigma \left(w_{\alpha }^{⊤}g+b_{\alpha }\right)$$

where *ϕ* is a hidden activation (e.g., ReLU). The scalar $\alpha \left(x\right)\in [0,1]$ is interpreted as the degree of trust in the RF prior for sample *x*.

#### 5.2.3. Interpretable Fusion

The final probability fusion formula is shown in Equation (17):

(17) $$F(x)=F_{RF}(x)+(1-\alpha (x))\underset{\mathrm{residual}\;\mathrm{correction}}{\underset{︸}{\left[F_{E}(x)-F_{RF}(x)\right]}}$$

This design means the expert subnet only needs to learn the delta between the RF prior and the true posterior, rather than estimating the full decision boundary from scratch.

This fusion endows the model with two interpretable operating regimes:

- α(x) → 1 (high-confidence zone): the correction term vanishes and the model relies entirely on the RF prior, which typically occurs when hard-rule features (e.g., successful regex matching) are strong.
- α(x) → 0 (correction zone): the model suppresses the RF prior and applies the full expert correction, typically when the sample lies in the RF’s decision-boundary ambiguity band or exhibits systematic distributional bias.

Through this mechanism, the model achieves a smooth transition from rule-driven to data-driven decisions.

### 5.3. Theoretical Analysis

We derive two analytical results for HGRN: (i) an adaptive error bound showing why gated residual fusion outperforms static averaging (Proposition 1), and (ii) a cross-domain generalization bound explaining why freezing the RF prior promotes stability under distribution shift (Theorem 1). Proof sketches are provided alongside each proposition.

#### 5.3.1. Adaptive Fusion Error Bound

Let F_RF(x) and F_E(x) denote the outputs of the RF prior and expert subnet, and let α (x) in [0, 1] be the gate weight. Denote the expected 0–1 risk by epsilon(cdot).

**Proposition** **1.** *The HGRN risk is bounded by the RF baseline plus a gated residual correction, as shown in the specific Equation (18):*

(18) $$epsilon\_HGRN\leq epsilon\_RF+E\left[\left(1-alpha\left(x\right)\right)|F\_E\left(x\right)-F\_RF\left(x\right)|\right]$$

The bound contains two terms: (i) epsilon_RF, the baseline risk of the frozen random forest, and (ii) a residual correction scaled by (1 − α(x)). When the gate fully trusts the RF prior (α → 1), the correction term vanishes and the bound collapses to the RF risk alone. When the gate distrusts the RF and triggers expert intervention (α → 0), the bound is governed by the pointwise divergence between the two predictors. Because the gate learns to activate correction only on samples where the RF is unreliable, HGRN adaptively selects the tighter per-sample bound rather than statically accumulating errors from both experts.

Proof sketch. For any sample x, a prediction error by F(x) implies one of two disjoint events: (i) $F_{RF}\left(x\right)$ errs, occurring with probability at most ϵRF; or (ii) $F_{RF}\left(x\right)$ is correct but the residual correction $\left(1-\alpha \left(x\right)\right)\Delta \left(x\right)$ pushes the prediction across the decision boundary. Because the gate learns to suppress the correction (α(x) → 1) precisely when the RF prior is reliable, the second term is active only on samples where the RF is unreliable. Decomposing the 0–1 risk over these two events and taking expectation yields the bound (18). The complete derivation and constant specification are given in Appendix D.

#### 5.3.2. Generalization Bound Under Distribution Shift

Let P_s and P_t be source and target distributions. Denote by D the H-divergence $d\_\left\{H\;delta\;H\right\}\left(P\_s,P\_t\right)$, and let $d\_VC=d\_VC^{RF}+d\_VC^{gate}$ denote the total VC-dimension of HGRN.

**Theorem** **1.** *For any F in H, the target error is bounded by Equation (19):*

(19) $$epsilon_{t}(F)<=epsilon_{s}(F)+D/2+{lambda}^{*}$$

where lambda^*^ is the ideal joint risk. Furthermore, with probability at least 1-delta, the empirical divergence hat D satisfies, as shown in the specific Equation (20):

(20) $$hat\;D\;<=\;D\;+\;C\;sqrt\left(d\_VC/N\_s\right)$$

where C is a universal constant.

Because the RF is frozen after source training, the active learnable parameters are restricted to the lightweight gating network, yielding a small d_VC. This tightens the bound and explains why pure neural baselines collapse under domain shift while HGRN exhibits more controlled degradation.

## 6. Experimental Evaluation

We evaluate the proposed HGRN on three real-world structured datasets to assess its effectiveness, robustness, and interpretability. We first describe datasets and baselines, then present overall performance, ablation studies, and rule effectiveness analysis.

### 6.1. Experimental Setup

#### 6.1.1. Datasets and Preprocessing

We use three datasets with markedly different distributions (Table 4).

CMS Open Payments. A U.S. healthcare payment dataset recording financial transfers to physicians and hospitals. As it lacks native sensitive-field labels, we establish a two-stage annotation protocol aligned with HIPAA Privacy Rule identifiers (45 CFR 164.514): (i) rule-based tagging of direct identifiers (physician name, NPI) and quasi-identifiers (zip code, specialty) using CMS documentation, and (ii) manual review by two annotators (inter-rater agreement κ = 0.91), with discrepancies resolved by a domain expert.

Home Credit. A Kaggle credit-risk dataset containing customer credit histories and behavioral features. Following GDPR Article 9 and China’s PIPL Article 28, fields are labeled sensitive if they pertain to identity, contact, financial status, or biometric/lifestyle indicators; non-sensitive fields include system-generated indices and temporal counters. Labels are assigned via rule-based classification cross-checked against the Kaggle data dictionary, with a 10% random sample manually verified.

Preprocessing is standardized across all datasets: values are cast to strings, missing entries filtered, and up to N = 100 non-empty values per column are sampled to compute entropy, texture, and uniqueness statistics. Zero-imputation fills any missing feature dimension, yielding a consistent six-dimensional vector per field.

#### 6.1.2. Baselines and Implementation Details

We compare against a diverse set of baselines, covering linear, deep, graph-based, and ensemble models:

Logistic Regression (LR)—linear baseline.

Pure MLP/Gated + Residual/Pure Attention—neural baselines.

GraphSAGE—graph-aggregation baseline (e.g., modeling relations between columns).

LightGBM—strong gradient boosting baseline.

FT-Transformer (Gorishniy et al., 2021) [26]—a deep-learning baseline for tabular data that embeds heterogeneous features and processes them through a Transformer encoder. We feed the same six-dimensional vector after normalization, using the default configuration (embedding dim 192, 3 Transformer blocks, 8 attention heads) with early stopping on a 10% validation split.

All models use the same feature inputs and evaluation protocol. For RF and LightGBM, we apply class-balancing strategies to mitigate label imbalance. For neural networks, we normalize inputs and use stable training configurations. Ablation experiments compare: Full Model (ours), RF only, Expert only, and a simple MLP baseline. Rule-effectiveness experiments evaluate each feature dimension in isolation. Complete implementation details—including the regex rule library (Table A1 and Table A2), domain lexicon (Table A3), BERT-NER configuration (Table A4), MI computation protocol (Table A5), and full hyperparameter settings (Table A6)—are provided in Appendix C to ensure reproducibility.

#### 6.1.3. Evaluation Metrics

To ensure statistical reliability while accommodating dataset-scale diversity, we adopt differentiated evaluation protocols, summarized as follows:

- Quantitative performance experiments (Section 6.2 and Section 6.3.1).
  DeSSI and HomeCredit employ stratified 5-fold cross-validation repeated over 5 independent runs with different random seeds (42, 123, 456, 789, 2024), reporting mean ± standard deviation.
  CMS employs single-run Leave-One-Out Cross-Validation (LOOCV) to maximize training-data utilization under extreme sample scarcity (N = 65 fields); consequently, CMS results report deterministic single-run values without cross-run variance.
- Qualitative interpretability analysis (Section 6.3.2).
  To obtain sufficient out-of-sample predictions for reliable density estimation of the gating weights, all three datasets—including CMS—adopt 5-fold CV for the α-distribution visualization only. This visualization protocol differs from the LOOCV protocol used for CMS quantitative evaluation.

Statistical significance is assessed via paired *t*-test: for DeSSI and HomeCredit, across the 5 independent runs; for CMS, across the per-field predictions of the representative LOOCV run (seed = 42).

### 6.2. Overall Performance

Observing the experimental results in Table 5, neural and graph baselines relying solely on hand-crafted features perform comparably (Macro-F1 0.75–0.77 on DeSSI), indicating that complex architectures do not automatically improve detection when feature expressiveness is limited. LightGBM substantially outperforms these (0.9338 on DeSSI), confirming the advantage of tree ensembles. FT-Transformer, a recent deep-learning architecture for tabular data, achieves strong in-domain results on DeSSI (0.9213) and HomeCredit (0.8442), validating Transformer-based feature interaction modeling when sufficient data is available. However, its performance narrows on CMS (0.7544), where extreme sample scarcity limits attention-based aggregation, and it trails HGRN on all datasets. HGRN achieves the best results across all three datasets: 0.9408 on DeSSI, 0.8164 on CMS, and 0.8535 on HomeCredit. The absolute gain is most pronounced on CMS (+0.0620 over FT-Transformer and +0.1288 over LightGBM), while on HomeCredit the margins tighten (+0.0093 and +0.0465, respectively) as stronger baselines already capture much of the structure.

### 6.3. Ablation and Gating Interpretability

#### 6.3.1. Module Contribution (Ablation Studies)

**Figure 3:**
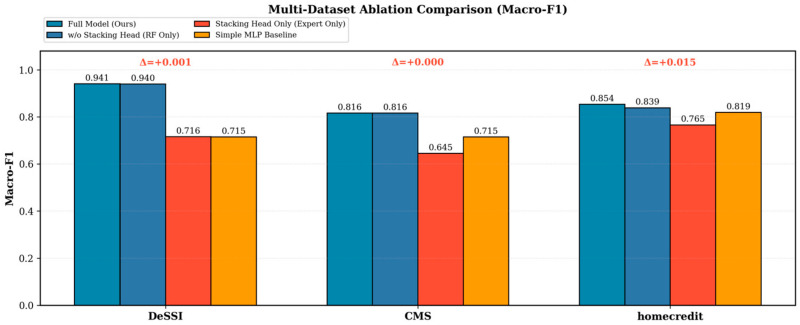
Multi dataset ablation comparison chart.

**Figure 4:**
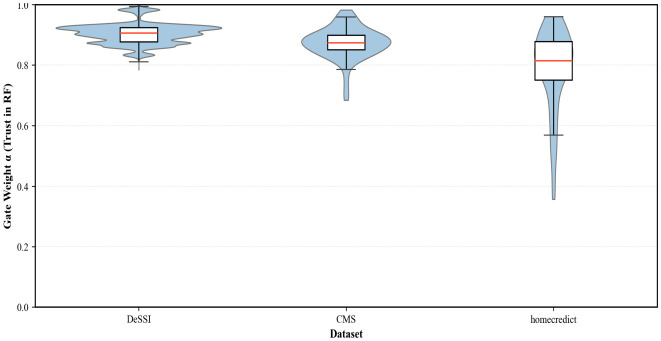
The distribution of different datasets. Gate weight distribution across datasets (α). Violin plots show the density of gating weights α ∈ (0, 1) for DeSSI, CMS, and HomeCredit. Blue shading indicates the kernel density estimate; the horizontal red line marks the median; the black box denotes the interquartile range; and the black whiskers extend to the minimum and maximum values within 1.5× the interquartile range. Wider sections indicate higher probability density. Higher α values indicate stronger trust in the RF prior.

Table 6 and Figure 3 reports the ablation results under the protocols defined in Section 6.1.3. On DeSSI, the Full Model achieves Macro-F1 = 0.9408 ± 0.0009, marginally improving over RF-only (0.9397 ± 0.0010, *p* = 0.19), indicating that the RF prior already captures most discriminative structure. On CMS, the Full Model and RF-only are tied at 0.8164 under LOOCV; extreme data scarcity (N = 65) forces the gate to default to near-complete trust in the RF prior (median α > 0.90), an intentional conservative behavior that avoids unreliable expert correction. On HomeCredit, the Full Model significantly outperforms RF-only (0.8535 ± 0.0138 vs. 0.8385 ± 0.0184, *p* < 0.05), with the most pronounced α long-tail (Figure 4), validating active expert involvement in complex financial fields. This domain-dependent spectrum—DeSSI anchoring, CMS conservatism, HomeCredit engagement—demonstrates adaptive rather than static fusion.

#### 6.3.2. Gating Weight Distributions

To explain the gain source of the Full Model, we analyze the gate weight α ∈ (0, 1), where larger values indicate stronger trust in the RF prior. For reliable density estimation, α values are collected from out-of-sample predictions under 5-fold CV (pooling all test folds), which differs from the LOOCV protocol in Table 6 for CMS/HomeCredit; we have verified that the qualitative patterns remain consistent across protocols.

As shown in Figure 4, the α distribution of DeSSI is generally closer to the high-value range and more concentrated, indicating that the discrimination rules of RF are more stable and reliable in this data domain, and the gating tends to “trust RF”. In contrast, the α distribution of CMS, especially HomeCredit, is more dispersed and exhibits a more pronounced long tail, indicating that the model triggers the “correction mode” more frequently on these datasets, reducing its dependence on RF in some fields and instead relying on higher-level expert networks for calibration. This trend is consistent with the ablation results showing more pronounced improvement on heterogeneous real-world domain datasets (in-domain), indicating that the gain of the Full Model is not a random increase caused by simple integration, but rather a differentiated fusion strategy learned by the gating mechanism in different domains.

**Figure 5:**
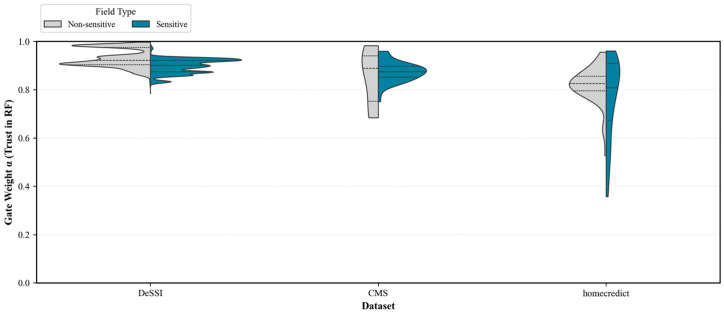
Differences in α distribution between sensitive and non-sensitive fields.

Figure 5 further illustrates the difference in α distribution between sensitive and non-sensitive fields. Overall, the weight distribution of the two types of fields does not completely overlap, indicating that the gate control output is not a constant, but has category dependent adaptability: in some datasets, the α of sensitive fields tends to be higher in the high-value range (more dependent on RF rules), while in data domains with more complex distributions, the difference in α between the two types of fields is more obvious and fluctuates more, reflecting that gate control can dynamically adjust the balance between “rule discrimination” and “learning correction” based on field types and feature patterns. This phenomenon provides interpretability support for the method proposed in this article: the model indeed learns different credibility allocation strategies on different field categories, thereby improving overall recognition performance and robustness.

**Figure A1:**
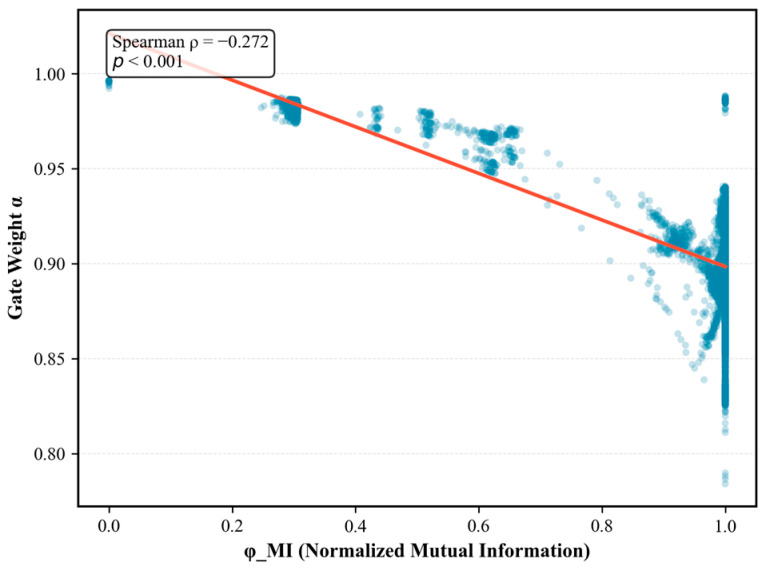
φ_MI versus gate weight α on the DeSSI test set. Each point denotes one field-level sample pooled from 5-fold CV. The red line shows the fitted regression trend. Spearman ρ = −0.272, *p* < 0.001, indicating that fields with stronger statistical dependence on known sensitive columns receive lower gating weights, thereby routing the sample toward the expert correction branch.

On DeSSI, the overall α distribution concentrates in the high-trust region (median > 0.90), reflecting that the RF prior already captures most discriminative structure in this domain. Nevertheless, fields with elevated mutual information exhibit measurable α suppression (Spearman ρ = −0.27, *p* < 0.001, Figure A1), confirming that the gate retains operational sensitivity to statistical-dependence signals even when the baseline prior is strong. Full correlation statistics and visualizations are provided in Appendix A.

### 6.4. Effectiveness of Individual Detection Rules

Observing Table 7, it can be concluded that:

Regex, MI, uniqueness, and fuzzy header matching exhibit comparable performance with Macro-F1 ≈ 0.43–0.47. While they achieve moderate recall (~0.51–0.66), their overall F1 is limited, reflecting narrow coverage and substantial errors.

NER alone performs worse, consistent with short, context-poor field values.

Statistical fingerprints are the strongest single feature (Macro-F1 ≈ 0.79), confirming that distributional patterns are highly informative for identifying sensitive fields.

Yet HGRN achieves Macro-F1 ~0.94, outperforming the best single feature by ~0.15. Practically, this means fewer missed implicit sensitive fields (e.g., hashed IDs) that single-signal detectors overlook, and less false-positive masking overhead—directly reducing both dark-data risk and compliance cost. The gain thus originates from complementary signal fusion rather than any single rule.

### 6.5. Threshold and Error Trade-Off Analysis

**Figure 6:**
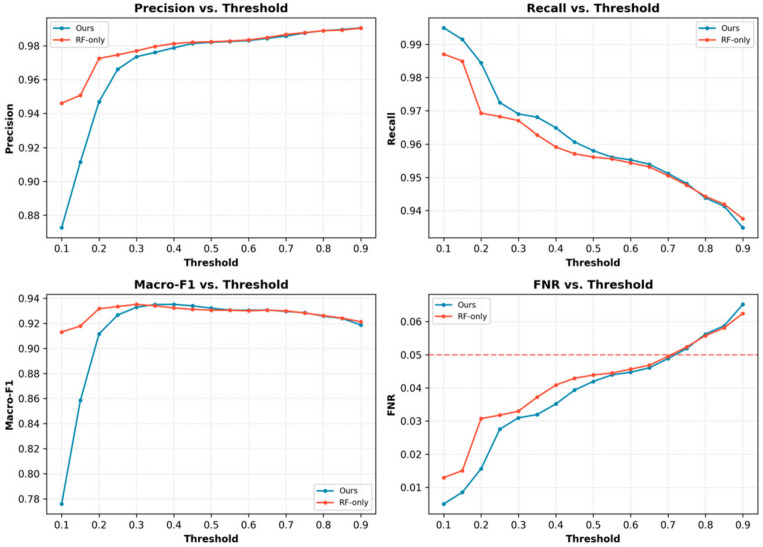
Threshold sweep analysis on DeSSI.

**Figure 7:**
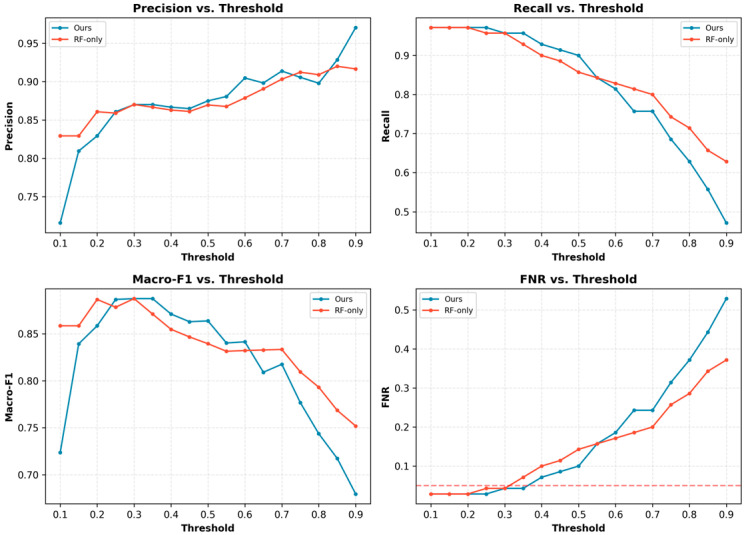
Threshold sweep analysis on HomeCredit.

**Figure A2:**
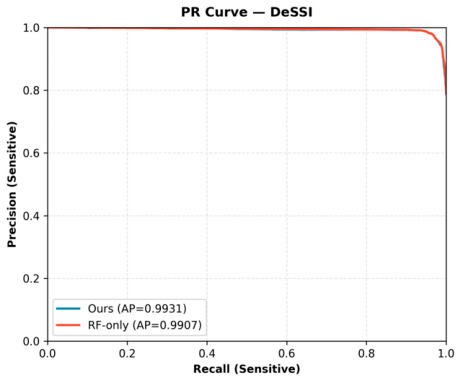
Precision-Recall curve on DeSSI. The Full Model achieves AP = 0.9931, marginally higher than RF-only (AP = 0.9907).

**Figure A3:**
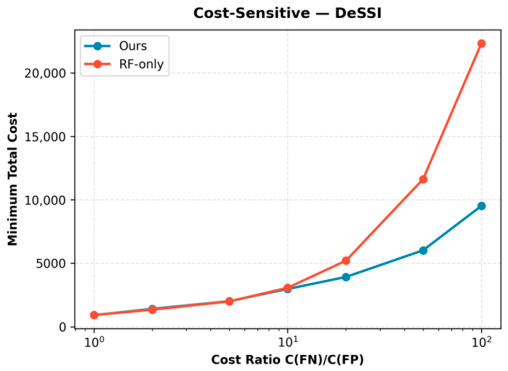
Cost-sensitive total cost on DeSSI under varying FN/FP cost ratios. When the cost of a missed sensitive field exceeds 10× that of a false alarm, the Full Model incurs substantially lower total cost than RF-only.

To assess the application-level trade-off between over-detection and missed detection, we conduct a threshold-sweep analysis on DeSSI and HomeCredit (Figure 6 and Figure 7). On DeSSI, lowering the threshold from 0.5 to 0.3 reduces FNR from 4.11% to 2.3%, directly supporting high-security governance scenarios where missed sensitive fields (“dark data” channels) must be minimized. On HomeCredit, HGRN maintains lower FNR than RF-only across the 0.3–0.7 threshold range, validating its operating-point robustness on moderate-scale datasets. Precision-Recall curves and cost-sensitive total-cost analysis are provided in Appendix B as supplementary validation (see Figure A2 and Figure A3).

### 6.6. Cross-Domain Transfer Evaluation

To evaluate behavior under strict distribution shift, we adopt a frozen-model stress-test protocol: all models are trained on DeSSI, then frozen and evaluated on target domains without parameter updates. LightGBM is allowed only decision-threshold calibration on a small target validation subset; neural baselines and HGRN parameters remain fixed. This protocol intentionally isolates the inherent generalization gap of structured sensitive-field detection, where weak semantics and heterogeneous naming conventions make frozen-model transfer inherently difficult.

Table 8 reports frozen-model transfer results. On HomeCredit, HGRN achieves 0.448 ± 0.026, higher than LightGBM (0.397 ± 0.011) and all pure neural baselines (0.297–0.353). On CMS, HGRN reaches 0.471 ± 0.012, exceeding LightGBM (0.339 ± 0.013) and neural baselines (F1 < 0.13). While absolute F1 scores remain modest—reflecting the fundamental difficulty of frozen-model transfer across weak-semantic structured data—HGRN consistently outperforms comparably constrained baselines.

Pure neural baselines collapse catastrophically and unpredictably: on CMS (65 fields, 84.6% sensitive) they scatter to F1 0.085–0.123, while on HomeCredit (121 fields, 24.8% sensitive) they uniformly degrade to 0.297–0.353. This confirms that frozen source-domain neural weights map target features to uninformative output regions regardless of architecture.

HGRN mitigates—though does not eliminate—this collapse because its frozen RF prior preserves structural knowledge that remains partially informative across domains (e.g., entropy patterns, regex anchors). In frozen-model transfer, the gate conservatively suppresses the expert branch (α → 1) when target-domain statistics are unavailable for reliable residual estimation, so the model remains anchored by the robust prior rather than degraded by neural components. Even on the extremely small CMS domain, HGRN averts the complete failure observed in pure neural networks. The interpretable gate weight α additionally provides explicit trust allocation between the rule-based prior and the data-driven expert, offering transparency when the model operates outside its training domain.

## 7. Conclusions

As the digital economy advances, secure circulation of data as a production factor is becoming a prerequisite for unlocking its full value. However, the weak semantics and distributional heterogeneity of structured data pose a dilemma for traditional sensitive field detection: over-detection hinders business flows, while missed detection creates dark data risks. To address the cross-domain generalization bottleneck, we presented an adaptive detection framework that combines multi-view complementary features with a hierarchical gated residual network.

At the feature level, we move beyond single-rule heuristics and construct a six-dimensional full-spectrum representation, spanning explicit rules (regex and header semantics), implicit statistical fingerprints (entropy, texture, uniqueness), and contextual association semantics (NER and mutual information). This design effectively fills semantic gaps in heterogeneous structured data. At the decision level, we introduce a dual-stream architecture that couples a robust RF prior with a learnable gating-and-expert correction layer. This “robust prior + dynamic calibration” mechanism explicitly resolves the cognitive conflict between hard rules and statistical distributions, enabling smooth adaptation between rule-dominated and complex, variant-rich regimes.

Extensive experiments on DeSSI, CMS Open Payments, and Home Credit show that the proposed method achieves a Macro-F1 of 0.9408 and significantly outperforms strong baselines including LightGBM and graph neural networks in in-domain settings. Under strict frozen-model cross-domain transfer, HGRN mitigates the catastrophic collapse suffered by pure neural baselines and retains non-trivial detection capability, confirming the protective role of the frozen RF prior. Analysis of gating weights confirms high decision interpretability: the model tends to trust the RF prior when rule-like cues are strong and increasingly relies on expert correction in complex healthcare and financial contexts. The framework delivers strong in-domain technical robustness and mitigates catastrophic failure under domain shift, while helping clarify security boundaries, reducing dark data risks, and avoiding unnecessary over-control and compliance friction.

Overall, this work provides a precise, scalable, and interpretable front-end governance solution for data classification and grading in complex business environments, contributing a practical technical pathway for balancing “protection” and “circulation” of data. Future work will explore extending the framework to federated learning settings for cross-domain privacy-preserving detection and incorporating large language models to improve frozen-model semantic inference in long-tail business systems, thereby further strengthening the basis for a dynamic and secure data ecosystem.

Limitations. A limitation of the current evaluation is the extreme sample scarcity in the CMS dataset (N = 65 fields), which necessitated single-run LOOCV and precludes reporting cross-run variance. Consequently, CMS quantitative results should be interpreted as indicative rather than statistically definitive, and the strong performance on CMS requires further validation on larger healthcare datasets before broader generalization claims can be made. Future work will expand the evaluation to additional healthcare and financial datasets with richer field-level annotations.

## Figures and Tables

**Table 1:** Capability boundaries of existing methods.

Method Paradigm	Typical Success Scenarios	Failure Scenarios	Root Cause
Rule-based matching	Standardized names (e.g., email) and rigid formats (credit card, phone)	Heterogeneous naming (u_id vs. user_code), hashed/anonymized data, semantically opaque fields	Rigid patterns do not generalize OOD
Single statistical model (RF/LightGBM)	Large, stable distributions; rules can be encoded in features	Small-sample overfitting; systematic bias in rule-heavy regimes (e.g., mislabeling high-entropy strings as passwords)	Single inductive bias cannot handle conflicts
Single deep model (MLP/Attention)	Complex nonlinear patterns and feature interactions	High variance under limited labels; poor robustness to domain shift; lack of strong priors	Data hungry and unstable

**Table 2:** Design logic and complementarity of the six feature dimensions.

Layer	Dimension	Blind Spot Addressed	Complementary Role
Explicit constraint	Regex matching	Strongly formatted sensitive fields (e.g., credit card, email)	When regex fails, header semantics provide weak supervision
	Header semantic alignment	Naming heterogeneity (user_id → user identifier)	
Implicit distribution	Statistical fingerprints	Hashed IDs, random tokens, semantically opaque identifiers	“Last resort” when both rules and semantics fail
	Uniqueness ratio	Primary keys/ID numbers (high uniqueness, low length variance)	
Contextual association	Mutual information	Quasi-identifiers that are harmless in isolation but risky in combination	Captures combination leakage risk, compensates incomplete local semantics
	BERT-based contextual semantics	Short-text semantic disambiguation	

**Table 3:** Regular patterns for typical sensitive data types.

Rule Class	Regex Pattern	Semantic Meaning
credit card	Combined Visa/MC/AMEX patterns	12–19 digits
email	[^@ \t\r\n]+@[^@ \t\r\n]+\.[^@ \t\r\n]+	Standard email format
phone	^\s*(?:\+?(\d{1, 3}))?[-.(]*(\d{3})[-. )]*(\d{3})[-. ]*(\d{4})	North American (with intl, code)

Note: Symbols within regex patterns follow standard regular-expression syntax.

**Table 4:** The dataset used in this article.

Dataset Name	Rows	Columns (Fields)	Feature Types	Sensitive Rate
DeSSI	100	31,000	Mixed	48.0%
CMS Open Payments	50,000	65	Numeric/Categorical	84.6%
HomeCredit	307,512	121	Mixed	24.8%

**Table 5:** Reports Macro-F1 scores across models and datasets.

Model	DeSSI	CMS	HomeCredit	Avg (Macro-F1)
Logistic Regression	0.7648 ± 0.0002	0.6455	0.7999 ± 0.0109	0.7367
Pure MLP	0.7553 ± 0.0015	0.6455	0.8193 ± 0.0171	0.7400
Gated + Residual	0.7657 ± 0.0051	0.6610	0.8078 ± 0.0064	0.7448
Pure Attention	0.7680 ± 0.0004	0.6308	0.8229 ± 0.0158	0.7406
GraphSAGE	0.7633 ± 0.0055	0.6610	0.7980 ± 0.0172	0.7408
LightGBM	0.9338 ± 0.0006	0.6876	0.8070 ± 0.0188	0.8095
FT-Transformer	0.9213 ± 0.0008	0.7544	0.8442 ± 0.0122	0.8399
HGRN (Ours)	0.9408 ± 0.0009	0.8164	0.8535 ± 0.0138	0.8702

**Table 6:** Ablation experiment data sheet.

Dataset	Configuration	Accuracy	Precision	Recall	Macro-F1
DeSSI	Full Model (ours)	0.9593 ± 0.0006	0.9297 ± 0.0010	0.9532 ± 0.0011	0.9408 ± 0.0009
	RF-only baseline	0.9585 ± 0.0007	0.9277 ± 0.0009	0.9532 ± 0.0012	0.9397 ± 0.0010
	Expert-only	0.7581 ± 0.0069	0.7024 ± 0.0001	0.7920 ± 0.0001	0.7159 ± 0.0047
	Simple MLP	0.7578 ± 0.0019	0.7097 ± 0.0020	0.8010 ± 0.0031	0.7152 ± 0.0020
CMS	Full Model (ours)	0.9385	0.8164	0.8164	0.8164
	RF-only baseline	0.9385	0.8164	0.8164	0.8164
	Expert-only	0.8154	0.6232	0.7486	0.6455
	Simple MLP	0.8769	0.6818	0.7825	0.7149
HomeCredit	Full Model (ours)	0.8612 ± 0.0223	0.8664 ± 0.0222	0.8550 ± 0.0230	0.8535 ± 0.0138
	RF-only baseline	0.8365 ± 0.0105	0.8396 ± 0.0092	0.8320 ± 0.0123	0.8385 ± 0.0184
	Expert-only	0.7700 ± 0.0429	0.7941 ± 0.0321	0.7841 ± 0.0405	0.7654 ± 0.0447
	Simple MLP	0.8207 ± 0.0074	0.8282 ± 0.0033	0.8301 ± 0.0060	0.8190 ± 0.0076

**Table 7:** Analysis of the effectiveness of different recognition rules.

Dimension/Method	Accuracy	Recall	Precision	Macro-F1
Regular rules	0.4692	0.6632	0.6427	0.4681
NER	0.3932	0.5267	0.5219	0.3900
MI	0.4690	0.6566	0.6344	0.4675
Statistics of fingerprints	0.8405	0.8356	0.7679	0.7902
uniqueness	0.4696	0.6569	0.6345	0.4681
Fuzzy header	0.4492	0.5110	0.5076	0.4292
HGRN (Ours)	0.9593 ± 0.0006	0.9297 ± 0.0010	0.9532 ± 0.0011	0.9408 ± 0.0009

**Table 8:** Frozen-model cross-domain transfer Results.

Transfer Pair	Model	Macro-F1
DeSSI → HomeCredit	HGRN (Ours)	0.448 ± 0.026
	LightGBM	0.397 ± 0.011
	Pure MLP	0.297 ± 0.012
	Gated + Residual	0.332 ± 0.020
	Pure Attention	0.353 ± 0.019
DeSSI → CMS	HGRN (Ours)	0.471 ± 0.012
	LightGBM	0.339 ± 0.013
	Pure MLP	0.085 ± 0.002
	Gated + Residual	0.092 ± 0.015
	Pure Attention	0.123 ± 0.011

## Data Availability

Publicly available datasets were analyzed in this study. Dataset: https://www.kaggle.com/datasets/sensitivedetection/dessi-dataset-for-structured-sensitive-information (accessed on 12 May 2025). CMS Open Payments: https://academictorrents.com/details/88f6fff84d7c2a2769348ab4c2b0ecb318b43752 (accessed on 12 May 2025). Default Risk: https://www.kaggle.com/c/home-credit-default-risk/data (accessed on 12 May 2025).

## Appendix C. Implementation Details and Reproducibility Checklist

### Appendix C.1. Regex Rule Library

Regular Expression Processing Protocol. The detection pipeline applies the regex library R in the following strict order: (1) all sampled values are cast to strings and stripped of leading/trailing whitespace; (2) each value is tested against every rule r ∈ R in the order listed in Table A1; (3) a field-level regex feature is activated if the fraction of matching values exceeds threshold ηr = 0.5 (i.e., at least 50% of the N ≤ 100 sampled values must match). No external libraries beyond Python 3.8 re are used.

The regex library R used in Section 5.1.1 covers five strongly formatted sensitive types derived from U.S. and international regulatory patterns (HIPAA, GDPR, PCI-DSS). Table A1 lists the exact patterns, their semantic coverage, and the matching threshold ηr = 0.5 (i.e., at least 50% of sampled values must match).

**Table A1:** Complete regex rule library R for explicit pattern matching.

Rule Class	Regex Pattern	Semantic Coverage	Source Standard
SSN	^(?!666|000|9\d{2})\d{3}-?(?!00)\d{2}-?(?!0{4})\d{4}$	U.S. Social Security Number	SSA/IRS formatting
Credit Card	(?:4[0-9]{12}(?:[0-9]{3})?|(?:5[1-5][0-9]{2}|222[1-9]|22[3-9][0-9]|2[3-6][0-9]{2}|27[01][0-9]|2720)[0-9]{12}|3[47][0-9]{13}|3(?:0[0-5]|[68][0-9])[0-9]{11}|6(?:011|5[0-9]{2})[0-9]{12}|(?:2131|1800|35\d{3})\d{11}|(?:5018|5020|5038|56|57|58|6304|6759|6761|6762|6763|0604|6390)\d{8,15})	Visa, MasterCard, AMEX, Diners Club, Discover, JCB, Maestro	PCI-DSS PAN patterns
Email	[^@ \t\r\n]+@[^@ \t\r\n]+\.[^@ \t\r\n]+	Standard email address	RFC 5322 simplified
Phone	^\s*(?:\+?(\d{1,3}))?[-. (]*(\d{3})[-. )]*(\d{3})[-. ]*(\d{4})(?: *x(\d+))?\s*$	North American phone with optional extension	NANP (E.164 subset)
Sex/Gender	^[mf]$|^(male|female)$	Biological sex or gender identity	HIPAA demographic
Website	https?:\/\/(www\.)?[-a-zA-Z0-9@:%._\+~#=]{1256}\.[a-zA-Z0-9()]{1,6}\b([-a-zA-Z0-9()!@:%_\+.~#?&\/\/=]*)	HTTP/HTTPS URLs	RFC 3986 subset

Note: Symbols within regex patterns follow standard regular-expression syntax.

Auxiliary tokenization patterns. The following patterns are used during header and value preprocessing (Section 5.1.1 and Section 5.1.2) but are not part of the sensitive rule library R:

**Table A2:** Auxiliary tokenization patterns.

Pattern	Regex	Purpose
Lowercase alpha	[a-z]+	Header token decomposition
Uppercase alpha	[A-Z]+	Header token decomposition
Positive decimal	^\+?[0-9]+(,[0-9]+)*\.[0-9]+$	Numeric value classification
Negative decimal	^-[0-9]+(,[0-9]+)*\.[0-9]+$	Numeric value classification
Positive integer	\+?[0-9]+(,[0-9]+)*	Numeric value classification
Negative integer	-[0-9]+(,[0-9]+)*	Numeric value classification
Punctuation	[!”#&\’()*,-./:;+?\\[\\]^_{|}~]+`	Character texture vector (special symbols subset)
Unicode symbols (Sc)	[<currency symbols>]+	Character texture vector (currency symbols)

Note: Symbols within regex patterns follow standard regular-expression syntax.

### Appendix C.2. Domain Lexicon for Header Semantic Alignment

**Table A3:** Domain Lexicon for Header Semantic Alignment.

Category	Roots
Identity	user, usr, name, id, ident, ssn, social
Contact	phone, tel, mobile, cell, mail, email
Location	addr, address, loc, city, state, zip, post, lat, lng
Personal traits	birth, dob, age, sex, gender, race, ethnic, ethnicity, religion, martial, status
Family	spouse, children, child, relationship
Financial	card, credit, bank, account, acct, bal, salary, pay, income, tax, insurance, insur, benef
Security credentials	pass, pwd, secret, token, key, ip, mac
Occupation	occupation, musician
Other	numbers

Construction protocol. The lexicon was built by (i) extracting high-frequency header tokens from the DeSSI training split, (ii) filtering tokens with χ^2^ association to the sensitive label, and (iii) manual consolidation of plural forms and abbreviations. No external gazetteer was used.

### Appendix C.3. BERT-NER Configuration for Contextual Semantics

**Table A4:** BERT-NER implementation parameters.

Item	Setting
Pre-trained model	dslim/bert-base-NER
Task pipeline	Named Entity Recognition (ner)
Tokenizer	dslim/bert-base-NER (WordPiece, vocab size 30,522)
Aggregation strategy	simple (sub-word grouping into word-level entities)
Device	CUDA GPU (index DEVICE_ID, single-GPU inference)
Prompt template	“Field name: {h}. Sample values: {v1}, {v2}, {v3}.”
Sample count k	3 non-empty values per field
Aggregation rule	Majority vote over the k sampled values
Confidence threshold τbert	0.5
Sensitive entity categories Csen	PER, ORG, LOC, MISC (mapped from CoNLL-2003 schema)

### Appendix C.4. Mutual Information Operational Function

**Table A5:** MI computation protocol.

Item	Setting
Reference set S	{fj:yj = 1}, i.e., all confirmed sensitive fields in the current table
Target field	Any field X in the same table
Sampling for speed	If table rows > 2000, random sub-sample Nmi = 2000 rows with random_state = 42; otherwise full table
Discretization	Implicit: each distinct string value is treated as a categorical level (no explicit binning)
Joint distribution	Empirical co-occurrence table computed from the sampled rows
Normalization	Inorm(X;Y) = I(X;Y)/min(H(X),H(Y))
Final scalar ϕMI(X)	maxY ∈ SInorm(X;Y)
Fallback for empty/constant fields	If H(X) < 10 − 10 or fewer than 2 non-null pairs, ϕMI(X) = 0.0

During cross-validation, S is restricted to the training-fold sensitive fields only; per-fold MI is recomputed to prevent leakage. For frozen-model transfer (Section 6.6), S is initialized by the rule-based regex detector (Table A1), and iterative refinement is disabled to maintain the frozen-protocol.

### Appendix C.5. Hyperparameters and Thresholds

**Table A6:** Thresholds Table.

Parameter	Symbol	Value
Regex activation threshold	ηr	0.5
BERT confidence threshold	τbert	0.5
BERT sample count	k	3
Max sampled values per field (entropy/texture)	N	100
MI sub-sample size	Nmi	2000
MI random seed	—	42
RF trees	K	200
Expert hidden layers	—	32
Gate hidden layers	—	32
Learning rate	—	10^−3^

## Appendix D. Proofs

### Appendix D.1. Proof of Proposition 1

From the residual form Equation (17),

(A1) $$F(x)=F_{RF}(x)+(1-\alpha (x))\left[F_{E}(x)-F_{RF}(x)\right]$$

For any sample x, if F(x) errs, then either FRF(x) errs, or FRF(x) is correct but the residual correction pushes the prediction across the decision boundary. Hence

$$1\{F(x)\neq y\}\;\leq \;1\{F_{\mathrm{RF}}(x)\neq y\}+(1-\alpha (x))1\{F_{E}(x)\neq y,F_{\mathrm{RF}}(x)=y\}$$

Taking expectations yields bound (18):

$$\epsilon (F)\;\leq \;\epsilon_{\mathrm{RF}}+E_{x}\left[\left(1-\alpha (x))\delta (x\right)\right],$$

where δ(x) ∈ {0, 1} indicates whether the expert over-corrects a correct RF output. When α(x) → 1 the second term vanishes; when α(x) → 0 the bound reduces to the pointwise divergence between the two predictors.

### Appendix D.2. Proof of Theorem 1

By standard domain-adaptation theory [27] (Ben-David et al., 2006), for any hypothesis h, the target-error bound (19) decomposes into three terms: the source risk ε_s(F), the distribution divergence D, and the ideal joint risk λ*. In HGRN, the RF prior is frozen after source training; the only trainable parameters belong to the lightweight gating network (input dim 6, hidden dim 32) and the expert MLP. Consequently, the VC-dimension d_VC appearing in (20) is determined solely by these small networks, which is substantially smaller than that of end-to-end deep models. Substituting the empirical divergence D and the restricted d_VC into (19) and (20) yields the bound with probability at least 1−δ. The tight restriction on d_VC explains why HGRN exhibits controlled degradation rather than catastrophic collapse under domain shift.
